# Delayed diagnosis is associated with complications following invasive meningococcal disease in Australian adolescents and young adults

**DOI:** 10.1007/s15010-026-02827-6

**Published:** 2026-05-18

**Authors:** Mark McMillan, Hassen Mohammed, Jim Buttery, Margaret Angliss, Belinda Barton, Christopher C. Blyth, Suja M. Mathew, Morgyn S. Warner, Renjy Nelson, Rory Hannah, Naomi Runnegar, Helen Siobhan Marshall

**Affiliations:** 1 Vaccinology and Immunology Research Trials Unit, Women’s and Children’s Health Network, North Adelaide, Adelaide, SA 5006 Australia; 2 School of Medicine, College of Health, Adelaide University, Adelaide, SA Australia; 3 Department of Infection and Immunity, Monash Children’s Hospital, Melbourne, VIC Australia; 4 Department of Paediatrics, University of Melbourne, Melbourne, VIC Australia; 5 Faculty of Health, CHERI, The Children’s Hospital at Westmead, Southern Cross University, Lismore, NSW Australia; 6 Wesfarmers Centre of Vaccines and Infectious Diseases, Telethon Kids Institute, University of Western Australia, Perth, WA Australia; 7 School of Medicine, University of Western Australia, Perth, WA Australia; 8 Department of Infectious Diseases, Perth Children’s Hospital, Perth, WA Australia; 9 Infectious Disease Unit, Central Adelaide Local Health Network, Adelaide, SA Australia; 10 Infectious Diseases, Clinical Immunology and Allergy Division of Medicine Lyell McEwin Hospital, Adelaide, SA Australia; 11 Infection Management Services, Princess Alexandra Hospital and University of Queensland, Brisbane, Australia

**Keywords:** Invasive meningococcal disease, Meningococcal disease, Adolescents and young adults, Sequelae

## Abstract

**Purpose:**

This study described the presenting features, initial assessment, hospital care, and complications at discharge among Australian adolescents and young adults with Invasive meningococcal disease (IMD).

**Methods:**

We conducted a retrospective study of IMD cases aged 15–25 years admitted between 2005 and 2018 in ten Australian hospitals.

**Results:**

A total of 104 IMD cases were included; 95.2% were due to serogroup B, and the mean age was 19.3 years (SD 2.1). Initial assessment most commonly occurred in the emergency department following self-presentation/private transport (50.0%), followed by general practice (32.4%) and paramedic-led pre-hospital care (17.7%). The most common nonspecific presenting symptoms were headache (80.8%), vomiting or nausea (75.0%), and fever (72.1%), and category 3 was the most frequently assigned triage level (40.6%). Half of cases required ICU admission (50.0%), with a mean ICU stay of 2.8 days (SD 2.4). Compared with patients who self-presented to the emergency department, initial assessment by paramedics in the community was associated with higher odds of ICU admission (adjusted AOR 4.42, 95% CI 1.81 to 16.5). A delay of more than 1 day between symptom onset and first medical presentation was associated with higher odds of discharge with one or more complications (aOR 2.88, 95% CI 1.13 to 7.35). At discharge, 43.3% had at least one complication, most commonly neurological sequelae (55.6% of those with complications), and 2 cases (1.9%) died.

**Conclusion:**

Delayed presentation was associated with a higher risk of IMD complications, highlighting the importance of early assessment and timely management to improve outcomes.

**Clinical trial registration:**

Clinicaltrails.gov, NCT03798574, https://clinicaltrials.gov/study/NCT03798574.

## Introduction

Invasive meningococcal disease (IMD) is an uncommon yet rapidly progressive infection caused by the Gram-negative bacterium *Neisseria meningitidis*, and is associated with significant morbidity and mortality globally [1]. IMD often presents as septicaemia, with or without meningitis, and may result in long-term sequelae that contribute to significant disability among those who survive [1]. Although IMD can occur at any age, disease incidence is highest in early childhood and adolescence, with consistent peaks observed in children aged 0–4 years and young people aged 15–25 years across most high-income countries [2, 3]. In Australia, 143 cases of IMD were notified in 2023, representing a 14% increase from 2022 (127 cases), with 98% of infections being laboratory-confirmed. Serogroup B remains the predominant cause in Australia, accounting for 84% of reported cases [3].

Early clinical presentation of IMD is often nonspecific, with symptoms such as fever, headache, lethargy, and vomiting overlapping with those of self-limiting viral illnesses, contributing to delayed recognition and diagnosis. Classical meningeal signs, including neck stiffness and altered mental status, are frequently absent at initial assessment, further impeding early diagnosis and timely intervention [4]. Septicaemic IMD can progress rapidly, with shock and multi-organ failure developing in less than 24 h [5]. A purpuric, non-blanching rash is a key diagnostic feature but may appear late and is absent in some cases [5]. In a UK study of adolescents with IMD, 66% developed a rash on average 19 h after symptom onset [6]. Other early indicators, such as limb pain, pallor, cold peripheries, and mottled skin, often occur within 7 to 12 h and are more common in children and adolescents [6].

Given the rapid progression of IMD, prompt clinical assessment and triage are critical. Delays in antibiotic administration have been associated with increased morbidity and mortality [7].Case fatality rates range from 10 to 15% in adolescents and young adults [8]. During the acute phase, patients with IMD often require intensive care management. Survivors may experience a range of cognitive, psychosocial, and physical sequelae that vary in severity and impact health-related quality of life [9–11]. While most research focuses on childhood survivors, limited data exist for adolescents and young adults. In particular, there is limited contemporary evidence describing early clinical presentation, initial assessment pathways, and triage patterns in this age group, and how these factors relate to subsequent clinical outcomes such as intensive care admission and complications at discharge. Historical cohort studies report that up to 61% of young adult survivors experienced lasting sequelae, negatively impacting educational and occupational outcomes [11, 12]. This retrospective study aimed to describe initial triages and hospitalization outcomes of adolescents and young adults (AYA) with confirmed IMD, and to identify associations between presenting clinical characteristics and intensive care unit (ICU) admissions.

## Materials and methods

### Study design and setting

This study was a retrospective cohort study conducted as part of an IMD case-control study [13], which examined the long-term impact of IMD on AYAs in Australia. IMD cases were identified from hospital records at participating sites using clinical diagnostic codes. The study included ten hospitals across Australia, comprising Western Australia (*n* = 1), Victoria (*n* = 2), New South Wales (*n* = 2), Queensland (*n* = 1), and Adelaide (*n* = 4). The hospitals are major tertiary referral centers serving large populations, equipped with specialized services and ICU management. Medical professionals from each participating site audited hospital records.

### Participants and data collection

Eligible cases included AYAs aged 15 to 24 years and 11 months who were admitted to a participating hospital between January 2005 and December 2018. Children younger than 15 years were not included, as the study was specifically designed to examine IMD in the adolescent and young adult population. Cases were identified using ICD-10 codes A49.0 to A39.9 and ICD-9 code 036, with J15.8 as an additional identifier where applicable. All included cases were required to have a laboratory-confirmed diagnosis of IMD. Data were collected and securely stored in the Research Electronic Data Capture (REDCap) system [14]. A questionnaire was used to record symptoms, complications, clinical outcomes, disease management and sequelae (Appendix). Socio-demographic information, including age, sex and residence at the time of admission, was collected. Clinical outcomes included the place of first presentation, the patient’s triage category upon arrival at the emergency department, duration from symptom onset to emergency department arrival, the meningococcal serogroup type, and ICU admission details such as length of stay. Data were entered and securely stored in the Research Electronic Data Capture (REDCap) system. Cases were classified as septicaemia, meningitis, or mixed presentation by a physician (SM) based on the following definitions. (1) Septicaemia was defined as a positive blood culture or PCR for *N. meningitidis.* (2) Meningitis alone was defined as a positive CSF culture or PCR for *N. meningitidis*. (3) A mixed presentation was defined as either the identification of *N. meningitidis* in both blood and CSF, or as a positive CSF result accompanied by evidence of sepsis supported by SOFA score. Conversely, cases with *N. meningitidis* identified in blood but not in CSF were classified as having both sepsis and meningitis if they exhibited specific clinical features, such as neck stiffness, a positive Kernig’s sign, or seizures. Isolated confusion or reduced consciousness was not considered sufficient.

### Statistical analyses

Descriptive statistics were used to summarise the demographic and clinical characteristics of IMD cases. Continuous variables were reported as means with standard deviations (SD) or medians with interquartile range (IQR), as appropriate, while categorical variables were summarized as counts and percentages. Age at admission was categorized into three groups: 15–17 years, 18–21 years, and 22–25 years. The Index of Relative Socioeconomic Disadvantage (IRSD) [15] scores were categorized into quintiles according to cut-points for the study region, whereby SES quintile 1 was the most disadvantaged and SES quintile 5 the least disadvantaged. Initial patient presentation was classified according to the setting of first clinical assessment: paramedic assessment in the community (pre-hospital care), emergency department presentation following self or private transport, or assessment by a general practitioner. The triage at first presentation was also categorized into five urgency levels (1 being most urgent, 5 being least urgent). The timing from the onset of first symptoms to hospital presentation was categorized into four intervals: <1 day, one to less than two days, two to less than three days, and more than three days.

The primary outcome of interest was the presenting symptom profile of IMD in Australian adolescents and young adults, examined in relation to subsequent clinical outcomes including ICU admission and complications at discharge. Cases with complications were further characterized according to complication severity. Univariate and multivariable logistic regression analyses were performed to examine associations between socio-demographic and clinical characteristics and the risk of ICU admission, as well as the presence of one or more long-term sequelae or death, with results reported as odds ratios (ORs) and adjusted odds ratios (aORs) with corresponding 95% confidence intervals (CIs). Analyses examining associations between individual presenting symptoms and outcomes among cases with meningitis with or without sepsis were conducted using univariate logistic regression only, as sample size limitations precluded multivariable adjustment for these models. All statistical analyses were conducted using Stata version 17.

### Ethics approval

Ethics approval was provided by the Women’s and Children’s Health Network (WCHN) Human Research Ethics Committee (HREC), Sir Charles Gairdner Group, Monash Health, and Western Sydney Local Health District Human Research Ethics Committees [13]. Governance was also obtained at all other affiliated sites [13]. A data collection form was completed for eligible cases in South Australia and Western Australia under a waiver of consent, and enrolled consented cases in Victoria and New South Wales. The study was an investigator-led trial, funded by Pfizer.

## Results

A total of 104 IMD cases were included, with an equal gender distribution. The predominant serogroup identified was B, accounting for 95.2% of cases (*n* = 99). Individuals aged 18 to 21 years constituted the majority, representing 64% of the total IMD cases. The IMD cases were distributed across each Quintile of Disadvantage based on the IRSD. However, the number from the least disadvantaged quintile was more than double that of the other quintiles. Approximately three-quarters of IMD cases lived in major cities (Table 1).

**Table 1:** Participant characteristics

Characteristics	*N* (%) *N* = 104
State	
South Australia	78 (75.0)
Victoria	9 (8.6)
Western Australia	12 (11.5)
New South Wales	4 (3.9)
Qld	1 (1.0)
Age at admission	
15–17 years	26 (25.0)
18–21 years	67 (64.4)
22–25 years	11 (10.6)
Sex	
Male	52 (50.0)
Female	52 (50.0)
Remoteness area	
Major cities of Australia	75 (72.1)
Inner regional Australia	18 (17.3)
Outer regional Australia	6 (5.8)
Remote Australia	4 (3.9)
Very remote Australia	1 (1.0)
Index of relative socioeconomic disadvantage IRSD quintile	
1 (most disadvantaged)	12 (11.5)
2	18 (17.3)
3	16 (15.4)
4	18 (17.3)
5 (least disadvantaged)	40 (38.5)
Meningococcal serogroup	
B	99 (95.2)
C	2 (2.0)
W	1 (1.0)
Untypeable	1 (1.0)
Unknown	1 (1.0)

Most IMD cases first underwent clinical assessment in the emergency department (51/104, 50.0%), followed by medical practice (33/104, 32.4%) and paramedics (18/104, 17.7%). A combination of both meningitis and septicaemia was the most frequent clinical presentation (33/104, 34.7%), with isolated meningitis and septicaemia occurring at similar proportions (29/104, 30.5% each). Patients were most commonly assigned triage category 3 (40.6%). High-acuity presentations (categories 1 or 2) accounted for 45.3%, while 14.1% were assigned category 4; no patients were triaged as category 5. Hospital admission was generally short, with 91/102 patients (89.2%) hospitalized for less than two weeks; however, half of all cases required ICU admission (52/104, 50.0%). Among ICU admissions, the mean length of stay was 2.8 days (SD 2.4), indicating that while intensive care was frequently required, prolonged ICU stays were uncommon (Table 2).

**Table 2:** Clinical presentation and hospital length of stay

First presentation	*N* (%)
Paramedic	18 (17.7)
Emergency Department	51 (50.0)
General Practitioner	33 (32.4)
*Missing*	2
Triage categories	
1	13 (20.3)
2	16 (25.0)
3	26 (40.6)
4	9 (14.1)
5	0 (0)
Missing	40
Days between first symptoms and presentation	
< 1 days	15 (14.4)
1 to < 2 days	46 (44.2)
2 to < 3 days	22 (21.2)
> 3 days	21 (20.2)
Length of stay (weeks)	
< 1	55 (53.9)
1 to < 2	36 (35.3)
2 to < 3	8 (7.8)
3 to < 4	1 (0.98)
> 4 weeks	2 (1.9)
Missing	2
ICU admission	
Yes	52 (50.0)
No	52 (50.0)
Days length of stay ICU (mean SD)	2.8 (2.4)
Clinical presentations in IMD cases	
Meningitis and/or Septicaemia	33 (34.7)
Septicaemia	29 (30.5)
Meningitis	29 (30.5)
Suspected Meningitis	4 (4.2)
Missing	9
Outcome of admission	
Discharged without complications	53 (51.0)
Transferred to other hospital with unknown outcome	4 (3.9)
Discharged with ongoing complications or sequela	45 (43.3)
Inpatient death	2 (1.9)

The most common nonspecific presenting symptoms of IMD included headache (80.8%), vomiting/nausea (75%), fever (72.1%), and muscle ache/joint pain (56.7%). More specific signs of IMD, such as a non-blanching rash (58.7%), stiff neck (43.3%), and photophobia (40.4%), were also common at presentation (Table 3).

**Table 3:** Symptoms and clinical signs within the first 12 h of presentation (*n* = 104)

Symptoms (Patient-reported)	Number	%
Headache	84	80.8
Vomiting / nausea	78	75.0
Fever (> 37.5 °C) *	75	72.1
Muscle ache / joint pain	59	56.7
Photophobia	42	40.4
Lethargy	32	30.7
Confusion	29	27.9
Irritable / unsettled	21	20.2
Respiratory symptoms or breathing difficulty	18	17.3
Leg pain	10	9.6
Refusing food / drink	6	5.8
Clinical signs (observed on examination)		
Non-blanching rash	61	58.7
Neck stiffness	45	43.3
Ill appearance	19	18.3
Hypotension (< 90 systolic or < 40 diastolic)	15	14.4
Prolonged capillary refill (> 2 s)	5	4.8
Cold hands / feet	5	4.8
Focal neurological deficit	5	4.8
Kernig’s sign	7	6.7
Brudzinski’s sign	2	1.9
Shock	4	3.9
Unconscious	4	3.9
Responsive to pain only	2	1.9
Back rigidity	2	1.9
Seizures	1	1.0

**Fig. 1:**
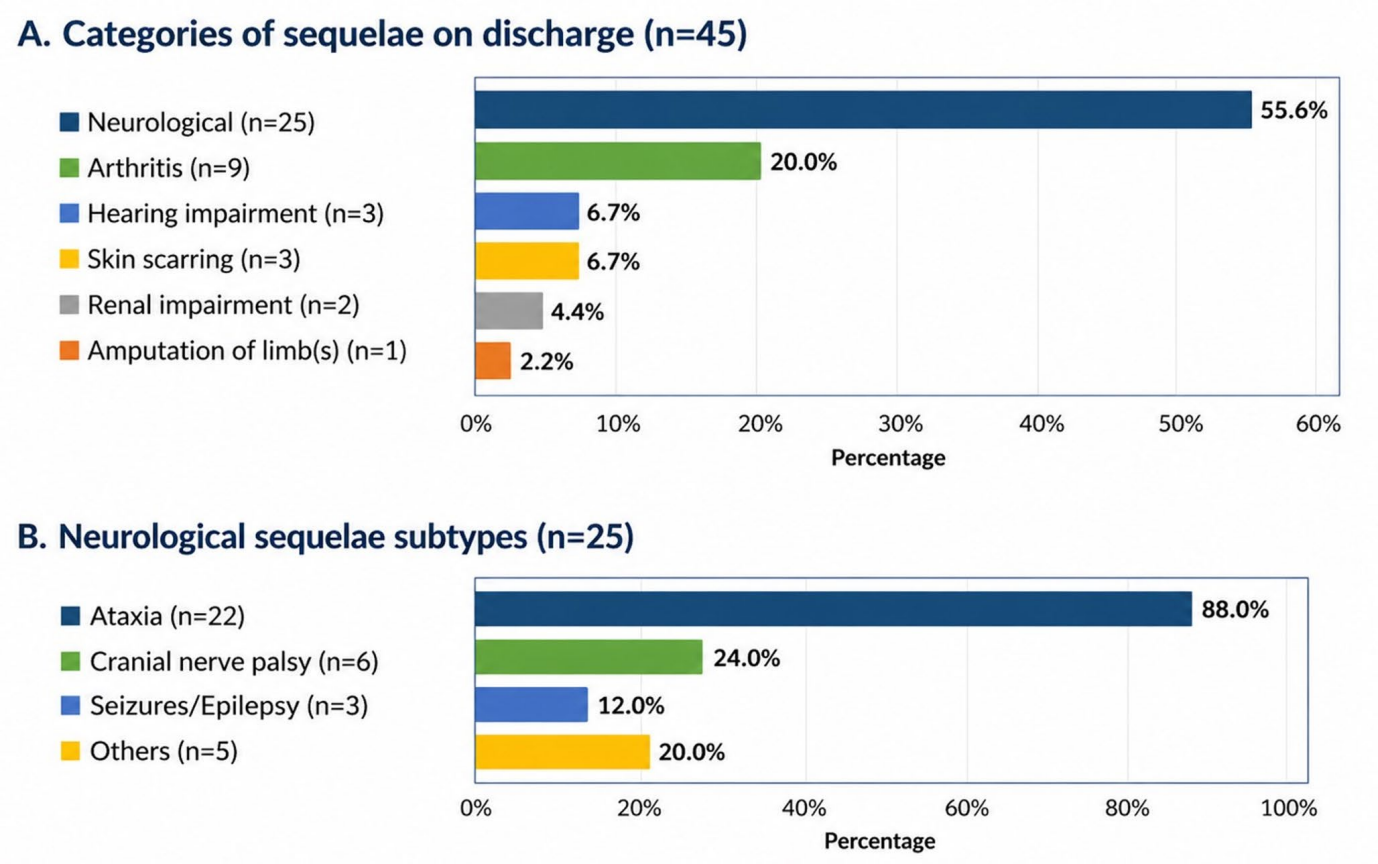
Distribution of complications at discharge among adolescents and young adults with invasive meningococcal disease

Almost half of IMD cases (*n* = 53, 51%) recovered and were discharged without complications, while 4 cases (3.9%) were transferred to other hospitals with unknown outcomes. Approximately 43% (45 cases) were discharged with ongoing complications, and there were 2 deaths, representing 2% of the total cases (Table 3). Among those with complications (*N* = 45), neurological complications were most common (25/45, 55.6%), with ataxia the most frequently reported neurological complication (22/25, 88.0%), followed by cranial nerve palsy (6/25, 24.0%) and seizures (3/25, 12.0%). Arthritis was the next most common complication, occurring in 9/45 cases (20.0%), while hearing impairment and skin scarring each affected 3/45 cases (6.7%), renal impairment occurred in 2/45 cases (4.4%), and amputation in 1/45 case (2.2%) (Fig. 1).

Compared with patients who self-presented to the emergency department or arrived via private transport, those initially assessed by paramedics providing pre-hospital care in the community had higher odds of ICU admission (aOR 4.42, 95% CI 1.81–16.5; *p* = 0.027). In contrast, general practitioner referral, which was associated with ICU admission in univariate analysis, was not independently associated after adjustment (aOR 1.17, 95% CI 0.42–3.27; *p* = 0.769). Patients assigned lower-urgency triage categories (≥ 3) had lower odds of ICU admission compared with those triaged as category ≤ 2 (aOR 0.19, 95% CI 0.05–0.66; *p* = 0.009). A delay of more than one day between symptom onset and first medical presentation was associated with increased odds of discharge with one or more complications (aOR 2.88, 95% CI 1.13–7.35; *p* = 0.027), while no other presenting characteristics were associated with complications at discharge (Table 4).

**Table 4:** Associations between presenting characteristics and ICU admission and discharge with ≥ 1 complication

Age at admission	ICU admission *n*/*N* (%)	Odds ratio (95% CI)	*P*-value	Adjusted odds ratio (95% CI) ^a^	*P*-value	≥ 1 complication *n*/*N* (%)	Odds ratio (95% CI)	*P*-value	Adjusted Odds ratio (95% CI)^a^	*P*-value
15–18 years	23/52 (44.2)	Reference				23/52 (44.2)	Reference			
18–25 years	29/52 (55.8)	1.60 (0.73 to 3.45)	0.243	1.83 (0.74 to 4.54)	0.190	24/48 (50.0)	1.26 (0.57 to 2.78)	0.566	1.40 (0.60 to 3.27)	0.435
Sex										
Female	29/ 52 (55.8)	Reference				22/49 (44.9)	Reference			
Male	23/ 52 (44.2)	0.63 (0.29 to 1.67)	0.243	0.53 (0.21 to 1.35)	0.182	25/51 (49.0)	1.18 (0.54 to 2.60)	0.681	1.00 (0.41 to 2.45)	0.991
Remoteness										
Major Cities of Australia	35/75 (46.7)	Reference				34/73 (46.6)	Reference			
Not a Major City	17/29 (58.6)	1.62 (0.68 to 3.87)	0.278	1.04 (0.30 to 3.55)	0.952	13/ 27 (48.1)	1.07 (0.44 to 2.59)	0.889	1.58 (0.51 to 4.85)	0.425
IRSD Quintile - Categories										
≤ 3	25/46 (54.3)	Reference				21/44 (47.7)	Reference			
≥ 4	27/58 (46.6)	0.73 (0.34 to 1.60)	0.432	0.56 (0.20 to 1.51)	0.250	26/56 (46.4)	0.95 (0.43 to 2.10)	0.898	1.15 (0.44 to 2.98)	0.771
First presentation										
ED	21/51 (41.2)	Reference				22/49 (44.9)	Reference			
GP	15/33 (45.5)	1.19 (0.49 to 2.89)	0.012	1.17 (0.42 to 3.27)	0.769	17/33 (51.5)	1.30 (0.54 to 3.17)	0.559	1.21 (0.43 to 3.40)	0.723
Paramedic	14/18 (77.8)	5 (1.43 to 17.4)	0.012	4.42 (1.81 to 16.5)	0.027	7/16 (43.8)	0.95 (0.30 to 2.99)	0.936	1.17 (0.37 to 3.67)	0.785
Triage categories ^b^										
1 to 2	19/29 (65.5)	Reference				13/28 (46.4)	Reference			
3 to 5	9/35 (25.7)	0.18 (0.06 to 0.54)	0.002	0.19 (0.05 to 0.66)	0.009	16/33 (48.5)	1.09 (0.39 to 2.99)	0.873	1.17 (0.38 to 3.67)	0.784
Days between first symptoms and presentation										
≤ 1 days	33/61 (54.1)	Reference				21/57 (36.8)	Reference			
≥ 1 day	18/41 (43.9)	0.67 (0.31 to 1.48)	0.323	0.58 (0.22 to 1.54)	0.271	25/41 (61.0)	2.62 (1.16 to 5.94)	0.021	2.88 (1.13 to 7.35)	0.027

^a^Adjusted odds ratios are mutually adjusted for all variables included in the model

^b^Triage category data were missing for 40 cases and were coded as “unknown”; missing values were not imputed

Among patients presenting with meningitis with or without sepsis, several symptoms were significantly associated with ICU admission. Altered mental status (OR 4.71, 95% CI 1.85–12.0), irritability or unsettled behaviour (OR 5.83, 95% CI 1.79–18.94), and hypotension (OR 18.8, 95% CI 2.34–150.7) were strong predictors of ICU admission. In contrast, neck stiffness was associated with reduced odds of ICU admission (OR 0.42, 95% CI 0.19–0.93). The classical triad of fever, neck stiffness, and altered mental status was observed in a minority of cases and was more prevalent among ICU-admitted patients compared with non-ICU patients (13.5% vs. 7.7%). None of the presenting features were significantly associated with the development of complications at discharge, although altered mental status (OR 1.94, 95% CI 0.80–4.67) and hypotension (OR 1.86, 95% CI 0.60–5.71) showed a non-significant trend toward increased risk. Among patients with sepsis-only presentations (*n* = 29), similar associations were observed, with hypotension and altered mental status associated with ICU admission. However, the small sample size limited statistical precision (Table 5).

**Table 5:** Relationship between presenting symptoms, ICU admission, and discharge outcomes (≥ 1 complication)

Meningitis +/- sepsis	ICU admission			DC with ongoing complications		
	Admitted *n*/*N* (%)	Not Admitted *n*/*N* (%)	Odds ratio (95% CI)	≥ 1 complication *n*/*N* (%)	No ongoing complication *n*/*N* (%)	Odds ratio (95% CI)
Neck stiffness	28/52 (53.9)	17/52 (32.7)	0.42 (0.19,0.93)	23/47 (48.9)	19/53 (35.9)	0.81 (0.37,1.81)
Fever	38/52 (73.1)	37/52 (71.2)	1.10 (0.46,2.60)	33/47 (70.2)	38/53 (71.7)	1.10 (0.46,2.60)
Change in mental status	24/52 (46.2)	8/52 (15.4)	4.71 (1.85, 12.0)	17/47 (36.2)	12/53 (22.6)	1.94 (0.80,4.67)
Triad of fever, neck stiffness, and change in mental status	7/52 (13.5)	4/52 (7.7)	1.87(0.51,6.85)	6/47 (12.8)	3/53 (5.7)	2.45 (0.57,10.43)
Hypotension (< 90 systolic or < 40 diastolic)	14/52 (27.0)	1/52 (1.9)	18.8 (2.34, 150.7)	9/47 (19.2)	6/53 (11.3)	1.86 (0.60,5.71)
Joint and muscle pain	28/52 (54.0)	31/52 (59.6)	0.79 (0.36,1.73)	24/47 (51.1)	34/53 (64.1)	0.58 (0.26,1.30)
Non-blanching rash	34/52 (65.4)	27/52 (51.9)	1.75 (0.79,3.86)	26/47 (55.3)	32/53 (60.4)	0.81 (0.37,1.81)
Lethargy	16/52 (30.8)	16/52 (30.8)	1 (0.43,2.31)	15/47 (31.9)	16/53 (30.2)	1.08 (0.46,2.54)
Irritable/unsettled	17/52 (32.7)	4/52 (7.7)	5.83 (1.79,18.94)	8/47 (17.0)	13/53 (20.8)	0.78 (0.28,2.16)
Respiratory symptoms/signs or breathing difficulty	8/52 (19.2)	10/52 (15.4)	0.76 (0.27,2.13)	9/47 (19.2)	7/53 (13.2)	1.56 (0.53,4.59)
Leg pain	4/52 (7.7)	6/52 (11.5)	0.64 (0.17,2.43)	4/47 (8.5)	6/53 (11.3)	0.73 (0.19,2.78)
Sepsis only						
Fever	12/17 (85.7)	12/17 (85.7)	1.5 (0.17,12.9)	12/14 (85.7)	12/14 (85.7)	1 (0.12,8.63)
Change in mental status	6/17 935.3)	0/12 (0.0)	NA	4/14 (28.6)	1/14 (7.1)	5.20 (0.48,56.4)
Hypotension (< 90 systolic or < 40 diastolic)	7/17 (41.2)	0/12 (0.0)	NA	5/14 (35.7)	2/14 (14.3)	3.33 (0.50,22.01)
Joint and muscle pain	8/17 (47.1)	10/12 (83.3)	0.18 (0.03, 1.10)	6/14 (42.9)	11/14 (78.6)	0.20 (0.04,1.11)
Non-blanching rash	12/17 (70.6)	7/12 (58.3)	1.71 (0.35, 8.31)	11/14 (78.6)	8/14 (57.1)	2.75 (0.51,14.9)
Lethargy	7/17 (41.2)	5/12 (41.7)	0.98 (0.21,4.51)	4/14 (28.6)	7/14 (50.0)	0.4 (0.08,1.96)
Irritable/unsettled	5/17 (29.4)	2/12 (16.7)	2.08 (0.32,13.6)	3/14 (21.4)	4/14 (28.6)	0.68 (0.12,3.95)
Respiratory symptoms/signs or breathing difficulty	2/17 (11.8)	1/12 (8.3)	1.47 (0.11,19.13)	1/14 (7.1)	1/14 (7.1)	1 (0.05,18.7)
Leg pain	1/17 (5.9)	2/12 (16.7)	0.31 (0.02,4.09)	1/14 (7.1)	2/14 (14.3)	0.46 (0.04,6.04)

## Discussion

This study describes the clinical presentation and early healthcare pathways of IMD in adolescents and young adults, including mode of presentation, triage allocation, and their association with ICU admission and discharge outcomes. Presenting features ranged from nonspecific symptoms, such as headache and fever, to more specific signs, including non-blanching rash and neck stiffness. These findings are consistent with previous reports demonstrating that early symptoms are often nonspecific and may contribute to delayed recognition and hospital presentation [5, 6].

Presentation may be different depending on the serogroup, with UK research showing that some adolescents during the most recent serogroup W outbreak presented with gastrointestinal symptoms mimicking gastroenteritis [16]. In this cohort, in which most cases were due to serogroup B, gastrointestinal symptoms were also relatively common, affecting 75% of participants. A recent outbreak of IMD due to serogroup B in the UK further highlights the ongoing risk among adolescents and young adults, with 20 confirmed cases all requiring hospitalization and two reported deaths [17]. IMD can rapidly escalate to life-threatening conditions, with most cases requiring hospitalization within 24 h of symptom onset.

Consistent with previous research [18], neurological complications on discharge were predominant, affecting approximately one-quarter of the total IMD cases and highlighting the need for follow-up and rehabilitation. Other physical sequelae, such as hearing impairment (3 cases), skin scarring (3 cases), and amputation (1 case), were less common compared to previous studies [18]. A UK study [12] among adolescents with IMD reported 3% required amputations and 18% had skin scarring. Another retrospective analysis of young adults who acquired IMD in college found that three out of 25 survivors underwent amputations, and one had extensive skin scarring [19]. Physical and neurological sequelae, like amputation, skin scarring, or hearing loss, are often more apparent and thus more likely to be reported. Half (50%) of the IMD cases in our study were admitted to the ICU, a higher rate compared to the 38% previously reported in an English study involving a similar age group of 15-24-year-olds [20].

Our study also found that patients arriving via ambulance had a significantly higher risk of ICU admission, likely reflecting more severe illness at presentation. Conversely, those presenting at emergency departments or through general practitioners showed a decreased likelihood of ICU admission. The association between altered mental status, hypotension, and ICU admission highlights the importance of recognizing early neurological and circulatory instability as indicators of severe disease in IMD. Although the classical triad of fever, neck stiffness, and altered mental status was observed more frequently among ICU patients, it was not significantly associated with ICU admission, consistent with prior studies showing that many IMD cases present atypically [4, 21]. These findings suggest that relying solely on classical meningeal signs may delay escalation of care, whereas attention to early signs of systemic compromise could improve triage and management decisions in emergency settings [4, 21].

Patients in rural or remote settings and those of lower SES were no more likely to require ICU admission or be discharged with complications than their urban and higher SES counterparts, indicating uniform care across different settings and socioeconomic levels. Similarly, there was no significant difference in ICU admissions or discharge outcomes between adolescents aged 15–18 and young adults aged 18–25, confirming consistent IMD care standards across these age groups.

Timely recognition and diagnosis of IMD remain vital, given the disease’s rapid progression and the potential for severe complications. Two systematic reviews [22, 23] found no evidence that pre-admission antibiotics for suspected cases of non-severe meningococcal disease reduce the risk of serious disease or death. This is thought to be due to methodological limitations in the available studies, which were largely observational and prone to diagnostic uncertainty and “indication bias,” where patients who were most severely ill were more likely to receive early antibiotics, thereby making outcomes appear worse in the treated group. Clinical guidelines in Australia [24] and the United Kingdom [25] continue to recommend the immediate administration of intramuscular or intravenous benzylpenicillin by ambulance paramedics when IMD is suspected, particularly if transfer times to hospital may be prolonged, given the potential for rapid progression.

Serogroup B, as the predominant serogroup in our study, remains the leading cause of IMD morbidity and mortality among adolescents and young adults in most high-income countries, including Australia. Several countries, including Australia, recommend the licensed 4CMenB vaccine to provide direct protection against MenB disease in adolescents [26]. Importantly, our study period preceded the South Australian state-funded 4CMenB vaccine program, launched in February 2019 for Year 10 students through the School Immunisation Program, with catch-up vaccination extended to young adults under 21 years of age [27]. As IMD can progress rapidly and may be fatal despite antibiotic treatment, vaccination remains the best strategy to protect high-risk individuals. Overall, 64% of IMD cases in our study occurred among young adults aged 18 to 21 years, emphasizing the importance of vaccinating adolescents before they enter a period of increased social interaction and behaviours that elevate IMD risk. More recently, state-funded infant and adolescent 4CMenB vaccine programs have also commenced in Queensland [28] and the Northern Territory [29], with a program recently announced in Tasmania.

Future research should identify barriers to timely hospital presentation and develop interventions, potentially using a prospective cohort design for more precise date on symptom onset and presentation. Larger cohorts are needed to discern patterns in early symptoms that predict IMD risk. This study has some limitations. Our descriptive analysis identified 104 IMD cases across four states, predominantly from South Australia, limiting its representativeness for the entire Australian population. Consequently, the demographic and clinical characteristics, as well as other outcomes of this study, may not be generalizable to all of Australia. In addition, the retrospective design and study period (2005–2018) may limit applicability to contemporary clinical practice, particularly in the context of evolving epidemiology and vaccination programs. This study was also restricted to adolescents and young adults aged 15 to 25 years and therefore does not describe IMD presentation or outcomes among children younger than 15 years.Additionally, missing data due to the retrospective case note design means that not all relevant variables could be captured, including the Sequential Organ Failure Assessment (SOFA) score, which may introduce bias or limit the accuracy of some analyses. Due to the sample size, we were limited to conducting univariate analyses when examining associations between presenting characteristics and ICU admission. As a result, the study was not able to determine independent associations.

In conclusion, the most common complication upon discharge involved ongoing neurological impairments, highlighting the critical need for follow-up and rehabilitation. Although the classical triad of fever, neck stiffness, and altered mental status was frequently observed among patients admitted to the ICU, it was not a reliable indicator of disease severity. A delay in diagnosis is associated with an increased risk of disease severity. These findings emphasize the importance of timely and accurate assessment at presentation to optimize patient outcomes and reduce the long-term impact of the disease.

## Data Availability

Deidentified individual participant data (including data dictionaries) will be made available, in addition to study protocols. The data will be made available to researchers who provide a methodologically sound proposal for use in achieving the goals of the approved proposal and pending approval from the WCHN HREC committee. Proposals should be submitted to helen.marshall@adelaide.edu.au.
